# Prognostic Role of Monocytes, Macrophages, and Lymphocytes in Diffuse Large B-Cell Lymphoma Patients Receiving R-CHOP: A Two-Year Survival Analysis

**DOI:** 10.12688/f1000research.168760.1

**Published:** 2025-08-22

**Authors:** Faisal Syarifuddin, Anna Mira Lubis, Agnes Stephanie Harahap, Hamzah Shatri, Wulyo Rajabto, Imam Subekti, Rudy Hidayat, Sukamto Koesnoe

**Affiliations:** 1 Division of Hematology and Medical Oncology, Department of Internal Medicine, Faculty of Medicine, Universitas Indonesia, Rumah Sakit Dr Cipto Mangunkusumo, Central Jakarta, Jakarta, 10430, Indonesia; 2 Department of Pathological Anatomy, Faculty of Medicine, Universitas Indonesia, Rumah Sakit Dr Cipto Mangunkusumo, Central Jakarta, Jakarta, 10430, Indonesia; 3 Division of Psychosomatic and Palliative Medicine, Department of Internal Medicine, Faculty of Medicine, Universitas Indonesia, Rumah Sakit Dr Cipto Mangunkusumo, Central Jakarta, Jakarta, 10430, Indonesia; 4 Division of Endocrinology, Metabolism, and Diabetes, Department of Internal Medicine, Faculty of Medicine, Universitas Indonesia, Rumah Sakit Dr Cipto Mangunkusumo, Central Jakarta, Jakarta, 10430, Indonesia; 5 Division of Rheumatology, Department of Internal Medicine, Faculty of Medicine, Universitas Indonesia, Rumah Sakit Dr Cipto Mangunkusumo, Central Jakarta, Jakarta, 10430, Indonesia; 6 Division of Allergy Immunology, Department of Internal Medicine, Faculty of Medicine, Universitas Indonesia, Rumah Sakit Dr Cipto Mangunkusumo, Central Jakarta, Jakarta, 10430, Indonesia

**Keywords:** DLBCL, monocyte count, CD163, CD8, tumor microenvironment, prognosis

## Abstract

**Background:**

Diffuse large B-cell lymphoma (DLBCL) exhibits heterogeneous clinical outcomes, including variations in event-free survival (EFS). Tumor microenvironment (TME) components, particularly absolute monocyte count (AMC), tumor-associated macrophages (TAMs), and tumor-infiltrating lymphocytes (TILs) have been implicated in prognosis, although findings remain inconsistent. This study evaluates the prognostic value of AMC, TAMs (CD163), and TILs (CD8) on two-year EFS in DLBCL patients treated with R-CHOP.

**Methods:**

A retrospective cohort study of 108 DLBCL patients treated from January 2014 to March 2021 was conducted. AMC was obtained from peripheral blood, while CD163 and CD8 expressions were analyzed via immunohistochemistry. Associations with two-year EFS were assessed using hazard ratios (HR), and correlations between AMC and tissue immune markers were evaluated.

**Results:**

High AMC and CD163 expression were significantly associated with poorer two-year EFS (HR = 9.82 and 8.57; both p < 0.001), whereas elevated CD8 expression predicted better outcomes (HR = 0.13; p < 0.001). AMC positively correlated with CD163 (r = 0.577; p < 0.001) and negatively with CD8 (r = –0.599; p < 0.001).

**Conclusion:**

AMC and CD163 are negative prognostic markers, while CD8 is protective. AMC may reflect the immune profile of the TME and serve as a practical prognostic biomarker in DLBCL.

## Background

Diffuse large B-cell lymphoma (DLBCL) is the most prevalent subtype of non-Hodgkin’s lymphoma (NHL) among adults, accounting for 30–40% of cases. Despite its aggressive nature, the introduction of chemotherapy regimens comprising rituximab, cyclophosphamide, doxorubicin, vincristine, and prednisone (R-CHOP) has significantly improved outcomes in DLBCL patients. Several studies have demonstrated the efficacy of R-CHOP, with reported 2-year event-free survival (EFS) rates ranging from 39% to 44%. ^1– 3^

To enhance prognostic stratification in the rituximab era, multiple clinical and laboratory parameters have been evaluated, including the International Prognostic Index (IPI) ^3^ and cell-of-origin (COO) classification based on the Hans algorithm. ^4^ These tools have contributed to risk-based therapeutic decision-making but remain limited in fully capturing the biological heterogeneity of DLBC. ^4, 5^

Recently, increasing attention has been directed toward the role of the tumor microenvironment (TME) in influencing DLBCL prognosis. The TME comprises various cellular elements, including fibroblasts, endothelial cells, pericytes, mesenchymal stromal cells, and immune cells, which may exert either anti-tumor or pro-tumor effects depending on their functional polarization. ^5, 6^ Notably, tumor-associated macrophages (TAMs), particularly the M2 subtype marked by CD163 expression, are associated with tumor progression and immune evasion, while CD8+ tumor-infiltrating lymphocytes (TILs) are typically linked to anti-tumor immune responses. ^5, 7^

While CD163 and CD8 have shown potential as prognostic biomarkers in DLBCL, the findings remain inconsistent due to varying cutoff thresholds and methodological differences across studies. ^5– 8^ Additional studies have suggested that the spatial distribution and density of these immune cells within the tumor tissue may also influence their prognostic relevance, highlighting the complexity of TME interactions. ^9^

Peripheral absolute monocyte count (AMC) has also emerged as a potential biomarker for DLBCL prognosis. Elevated AMC has been associated with unfavorable survival outcomes, possibly due to its contribution to the immunosuppressive TME and support of malignant B-cell proliferation. ^10, 11^ However, contrasting evidence suggests that AMC at diagnosis may not reliably predict disease relapse or treatment response. ^12, 13^

Given the evolving interest in immunological markers, integrating peripheral and tissue-based immune parameters could provide a more comprehensive prognostic model for DLBCL. ^14^ Therefore, this study aims to assess the prognostic value of AMC, CD163-positive TAMs, and CD8-positive TILs on two-year EFS in patients with DLBCL receiving R-CHOP therapy.

## Methods

### Study design and setting

This retrospective cohort study analyzed medical record data from patients diagnosed with diffuse large B-cell lymphoma (DLBCL) at Dr. Cipto Mangunkusumo Hospital (RSCM) and the affiliated anatomical pathology laboratory between January 2014 and March 2021.

### Ethics statement

The study was approved by the Health Research Ethics Committee of the Faculty of Medicine, Universitas Indonesia (FKUI) and Cipto Mangunkusumo National General Hospital (RSUPN), under reference number KET-133/UN2.F1/ETIK/PPM.00.02/2023. All subjects provided written informed consent, and patient confidentiality was maintained throughout the study.

### Patient selection

Inclusion criteria were as follows: (a) age ≥18 years, (b) diagnosis of DLBCL according to World Health Organization (WHO) classification, (c) received initial R-CHOP chemotherapy regimen consisting of 6–8 cycles, with or without radiotherapy, and (d) completed chemotherapy with an average delay of no more than one week per cycle.

Exclusion criteria included: (a) history of indolent lymphoproliferative disorders, (b) discordant or multiple histopathological findings, (c) diagnosis of HIV infection, (d) primary central nervous system (CNS) DLBCL, (e) presence of terminal comorbidities (e.g., end-stage renal failure, decompensated liver cirrhosis, or advanced heart failure), and (f ) incomplete clinical or pathological data.

Patient status was confirmed via telephone contact using the numbers recorded in the medical charts. Baseline clinical characteristics collected included demographic information, number of extranodal sites, Ann Arbor stage, serum lactate dehydrogenase (LDH) levels, International Prognostic Index (IPI) score, and cell-of-origin classification based on the Hans algorithm.

### Peripheral blood analysis

Absolute monocyte count (AMC) was determined using peripheral blood samples analyzed via a chromatographic method with a Sysmex XN-1000 or XN-3000 hematology analyzer.

### Immunohistochemical staining

Formalin-fixed, paraffin-embedded (FFPE) tissue blocks were sectioned at 3–4 μm and mounted on poly-L-lysine-coated slides. Sections were deparaffinized in xylene and rehydrated through a graded alcohol series. Antigen retrieval was performed using Tris-EDTA buffer (pH 9.0) in a decloaking chamber at 98°C for 10 minutes, followed by cooling and PBS washing. Endogenous peroxidase activity was blocked using SeyTek Super Block. Slides were then incubated for 30 minutes with Bond™ ready-to-use primary antibodies CD163 and CD8 (Biocare Medical, Netherlands). After washing, slides were treated with a secondary Val Universal Linker and visualized using DAB high contrast substrate for 30 seconds. Counterstaining was performed using Mayer’s hematoxylin, followed by bluing in lithium carbonate and dehydration in graded alcohol and xylene. Coverslipping was performed with entellan. Negative controls were included for each staining batch, while tonsil tissue was used as a positive control. For analysis, five representative high-power fields (HPFs, 400× magnification, area 0.24 mm ^2^) were photographed using a Leica ICC50HD microscope with camera attachment. Quantification of CD163-positive macrophages and CD8-positive lymphocytes was performed using ImageJ ^®^ software. CD163 was reported as absolute counts, while CD8 was expressed as percentage of stained T cells per HPF. Intensity of staining was not assessed due to potential variability from tissue fixation. All evaluations were independently conducted by two blinded investigators to ensure inter-observer reliability.

### Statistical analysis

Optimal cutoff values for AMC, CD163, and CD8 were determined using receiver operating characteristic (ROC) curve analysis and area under the curve (AUC) evaluation. Correlations between AMC, CD163, and CD8 were analyzed using Spearman’s correlation test. Event-free survival (EFS) was analyzed using Kaplan–Meier curves and log-rank testing. Univariate and multivariate Cox proportional hazards regression models were employed to estimate hazard ratios (HRs) and 95% confidence intervals (CIs). Variables with *p* < 0.05 in univariate analysis were included in the multivariate model. A *p*-value of < 0.05 was considered statistically significant. All statistical analyses were conducted using SPSS version 20.0 (IBM Corp., Armonk, NY, USA). All data were verified by two independent reviewers to ensure accuracy and completeness.

## Results

### Patient characteristics

From January 2014 to March 2021, a total of 108 patients diagnosed with DLBCL and fulfilling the inclusion and exclusion criteria were included in the study. Nine patients had missing lactate dehydrogenase (LDH) data. Of the total subjects, 56 (52%) were male, and 72 (66.7%) were under the age of 60 years (median: 53 years; range: 18–88 years). Early-stage disease (Ann Arbor stage I–II) was observed in 60 patients (55.6%), and 89 patients (82%) had 0–1 extranodal lesions. Eighteen subjects (18.2%) had LDH levels below the upper normal limit (<1× ULN), while 75 (75.8%) had a low-risk International Prognostic Index (IPI) score (0–2). Based on Hans classification, the germinal center B-cell (GCB) subtype was identified in 33 patients (30.6%). Over the two-year follow-up, 58 patients (53.7%) experienced event-related outcomes, while four patients (3.7%) remained event-free. The median values of AMC, CD163, and CD8 were 619.2 cells/μL (IQR 455.0–759.45), 21.5% (IQR 14.25–27.0%), and 23% (IQR 14–39%), respectively. Detailed demographic and clinical data are summarized in Table 1.

**Table 1.** Demographic and clinical characteristics of study subjects.

Variable	Total (%)
**Gender** (n = 108)	
Male	56 (52)
Female	52 (48)
**Age** (Median 53 years, range 18-88 years)	
≤60 years	72 (66,7)
>60 years	36 (33,3)
**Ann Arbor Staging** (n = 108)	
I or II	60 (55,6)
III or IV	48 (44,4)
**Extranodal lesion** (n = 108)	
0-1	89 (82)
>1	19 (18)
**Serum Lactate Dehydrogenase (LDH)** (n = 99)	
≤1 x normal	18 (18,2)
>1 x normal	81 (81,8)
**IPI Score** (n = 99)	
Low risk (0-2)	75 (75,8)
High risk (3-5)	24 (24,2)
**Protein expression** (n = 108)	
CD8, Median (IQR)	0,23 (0,14-0,39)
CD163, Median (IQR)	21,5 (14,25-27,0)
**AMC** (n = 108), Median (IQR)	619,2 (455,0-759,45)
**Subtypes based on Hans algorithm** (n = 108)	
GCB	33 (30.6)
Non-GCB	75 (69,4)
**Events in 2 years** (n = 108)	
Yes	58 (53,7)
No	46 (42,6)
No Information	4 (3,7)

### Immunohistological characteristics and optimal cutoff values for AMC, CD163, and CD8

Receiver operating characteristic (ROC) analysis was performed to determine the optimal cutoff points for AMC, CD163, and CD8 in predicting 2-year event-free survival (EFS). The optimal AMC cutoff was 631 cells/μL, with an AUC of 0.923 (95% CI: 0.866–0.981), sensitivity of 82.8%, and specificity of 90.0%. For CD163 expression, the optimal cutoff was 23%, yielding an AUC of 0.931 (95% CI: 0.880–0.983), sensitivity of 75.9%, and specificity of 96.0%. CD8 expression had an optimal cutoff value of 27.5%, with an AUC of 0.852 (95% CI: 0.773–0.930), sensitivity of 84.5%, and specificity of 84.0%.

**Figure 1.**
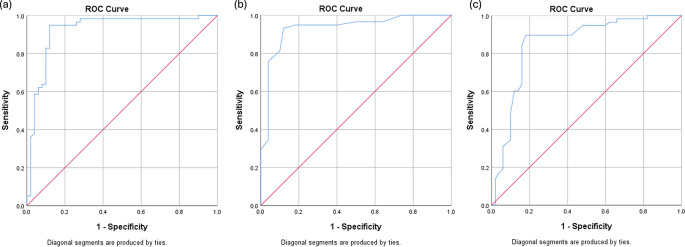
Quantitative ROC curves for 2-year Event-Free Survival: (a) AMC, (b) TAM CD163, and (c) TIL CD8.

**Figure 2.**
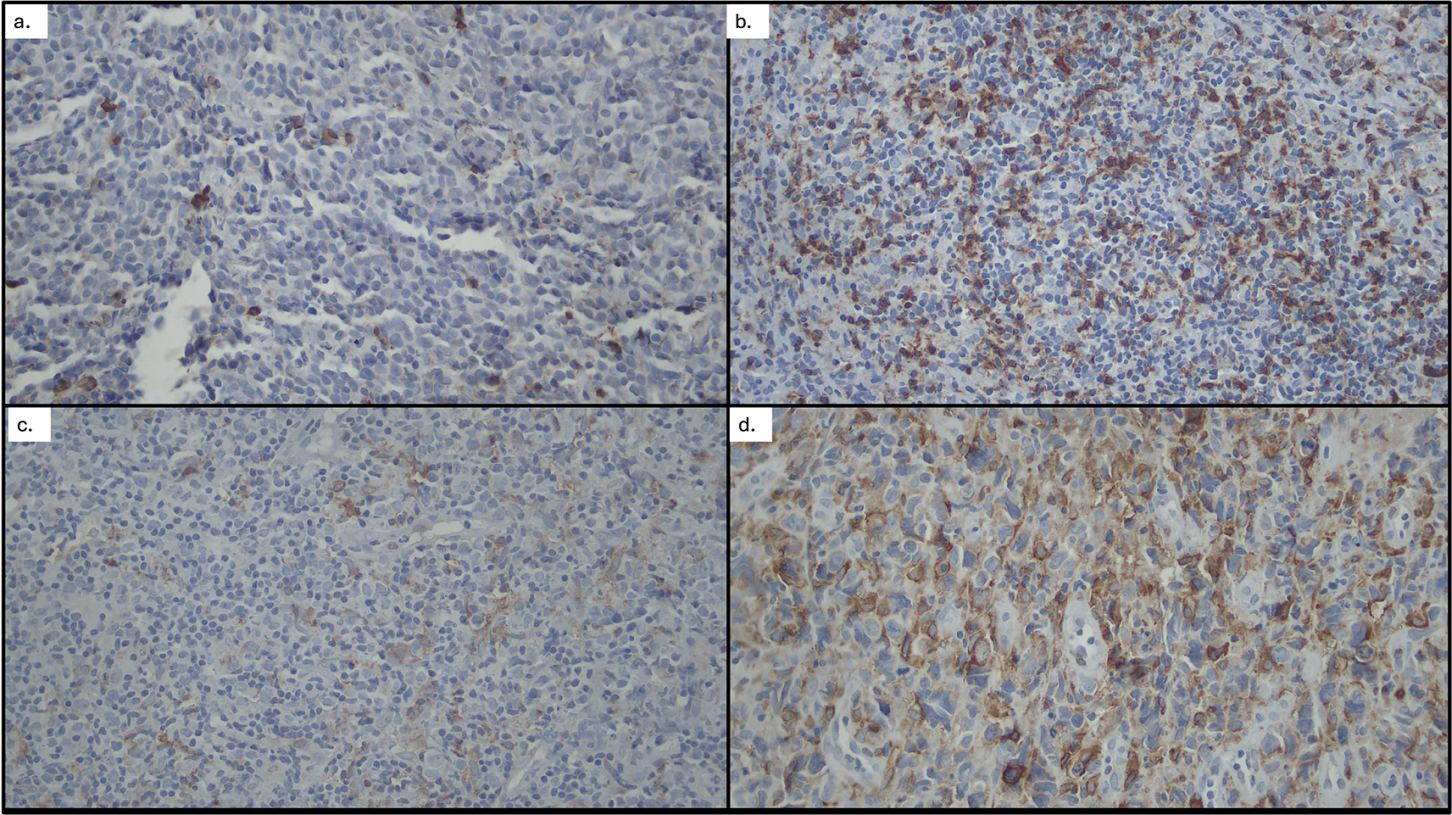
CD163 and CD8 expression in DLBCL tissues (100×): (a) Low CD163 expression, (b) High CD163 expression, (c) High CD8 expression, (d) Low CD8 expression.

ROC curves are illustrated in Figure 1. Patients were stratified into high- and low-expression groups based on these cutoff values ( Figure 2).

### Correlation between AMC and CD163 or CD8 expression

**Figure 3.**
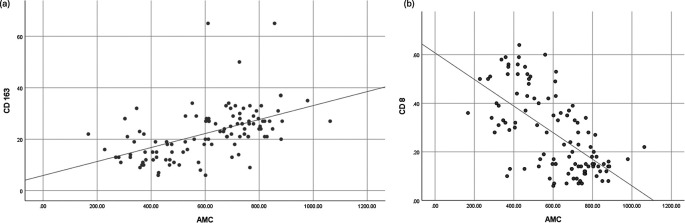
Correlation between (a) AMC and TAM CD163, and (b) AMC and TIL CD8.

Spearman’s correlation analysis demonstrated a significant positive correlation between AMC and CD163 expression (r = 0.577, *p* < 0.001), and a significant negative correlation between AMC and CD8 expression (r = -0.599, *p* < 0.001). These results suggest a possible immunological link between peripheral monocyte counts and the tumor immune microenvironment. Correlation plots are presented in Figure 3.

### Impact of AMC, CD163, and CD8 on 2-Year EFS

**Figure 4.**
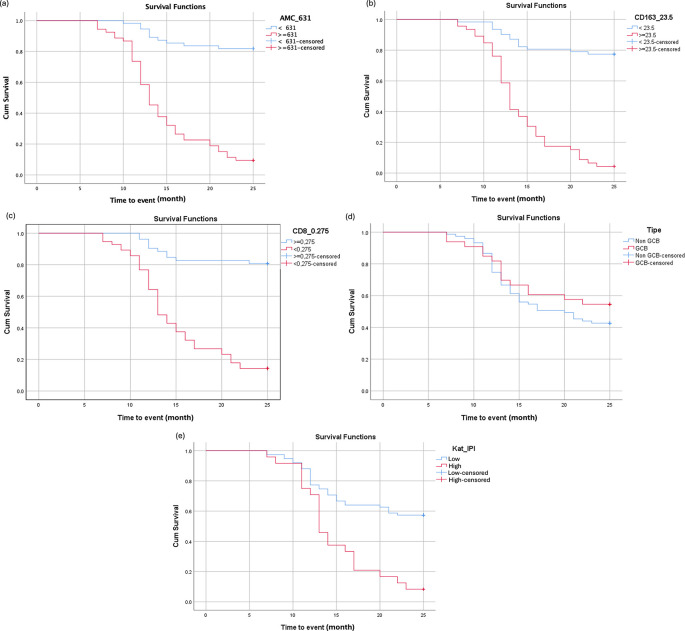
Kaplan-Meier survival curves for DLBCL patients based on (a) AMC, (b) TAM CD163, (c) TIL CD8, (d) COO, and (e) IPI score.

High AMC levels (≥631 cells/μL) were significantly associated with poorer 2-year EFS (HR = 9.82; 95% CI: 4.89–19.70; *p* < 0.001). Similarly, elevated CD163 expression (≥23%) was linked to worse survival outcomes (HR = 8.57; 95% CI: 4.58–16.05; *p* < 0.001). Conversely, patients with CD8 expression ≥27.5% had significantly better EFS outcomes. Subgroup analysis using the Hans classification algorithm revealed no significant difference in EFS between the GCB and non-GCB subtypes (HR = 0.749; 95% CI: 0.416–1.349; *p* = 0.336). However, a high IPI score was significantly associated with reduced EFS (HR = 3.06; 95% CI: 1.77–5.35; *p* < 0.001). Full statistical comparisons are shown in Table 2 and Figure 4.

**Table 2.** The relationship of different variables on patient survival.

Variable	Event		HR (CI 95%)	p
	Yes (%)	No (%)		
**AMC**				
≥631	48 (90,6)	5 (9,4)	9,817 (4,891-19,703)	<0,001
<631	10 (18,2)	45 (81,8)		
**CD163**				
>23	44 (95,7)	2 (4,3)	8,571 (4,576-16,053)	<0,0001
<23	14 (22,6)	48 (77,4)		
**CD8**				
≥0,275	9 (19,2)	48 (80,8)	0,128 (0.064-0.256)	<0,001
<0,275	48 (85,7)	8 (14,3)		
**GCB/nonGCB**				
GCB	15 (45,5)	18 (54,5)	0,749 (0,416-1,349)	0,336
nonGCB	43 (57,3)	32 (42,7)		
**IPI**				
High	22 (91,7)	2 (8,3)	3,075 (1,768-5,349)	<0,001
Low	32 (42,7)	43 (57,3)		

## Discussion

This study evaluated the prognostic value of absolute monocyte count (AMC), tumor-associated macrophages (TAM, CD163), and tumor-infiltrating lymphocytes (TIL, CD8) on the two-year event-free survival (EFS) of patients with diffuse large B-cell lymphoma (DLBCL) receiving R-CHOP chemotherapy. The median age of patients in our cohort was 53 years, consistent with previous studies conducted in Indonesia, but younger compared to cohorts in Western and East Asian countries such as Japan, Korea, and China, where median ages range from 63–64 years. ^2, 9, 14^ This difference may reflect underlying genetic polymorphisms, environmental exposures, and the prevalence of Epstein–Barr virus infection. ^15, 16^

Extranodal involvement was observed in 27% of patients, aligning with findings that a considerable proportion of DLBCL originates extranodally without overt nodal disease. ^16^ Furthermore, over half of the patients (55.6%) presented with early-stage disease (Ann Arbor stage I–II), a trend previously reported among Asian DLBCL cohorts. ^14^ This may be attributed to differences in diagnostic imaging; in Western populations, more frequent use of positron emission tomography (PET) may lead to more accurate staging and, consequently, higher stage classification. ^14, 16^

During the two-year follow-up, 58 patients experienced relapse, progression, need for additional therapy, or disease-related mortality. These findings are in line with previous literature suggesting that 40–50% of DLBCL patients experience treatment failure or recurrence. ^17, 18^

We identified optimal cutoff values for AMC (631/mm ^3^), CD163 (23/HPF), and CD8 (27.5%/HPF), which were predictive of EFS. The median survival time of 13 months in our cohort supports findings from earlier studies showing worse outcomes in patients with AMC > 630/mm ^3^. ^11, 19^ Similarly, the prognostic significance of high CD163 expression is consistent with the study by Li et al., which identified a cutoff of 19/HPF for CD163-positive TAMs and associated high expression with poorer progression-free and overall survival. ^19– 21^

On the other hand, patients with CD8+ TIL expression ≥27.5% had significantly improved survival, corroborating earlier studies that found CD8+ infiltration to be beneficial. ^7^ Although our cutoff threshold was higher than those used in previous studies (e.g., CD8 ≥5%), this discrepancy may reflect differences in tumor location, histological techniques, or scoring methodology. In particular, the use of whole-tumor section analysis rather than tissue microarrays may contribute to variability in TIL quantification across studies. ^22– 24^

A moderate positive correlation was observed between AMC and CD163 expression, consistent with prior findings by Li et al. ^19, 25^ In contrast, a negative correlation between AMC and CD8 expression was identified, which diverges from findings in other malignancies, such as breast cancer, where a weak positive correlation was reported. ^19, 26^ This highlights the context-dependent role of monocytes and lymphocytes in different tumor microenvironments.

The cell-of-origin (COO) classification using the Hans algorithm did not significantly impact 2-year EFS (p = 0.336), aligning with several studies that have questioned its prognostic reliability. ^20, 27^ This may be due to the algorithm’s limitations in accounting for clinical heterogeneity and its reliance on immunohistochemistry rather than molecular profiling. Moreover, it excludes important prognostic factors such as age, disease stage, and comorbidities. ^21, 28^

In contrast, the International Prognostic Index (IPI) remained a significant predictor of outcome, with low IPI scores associated with significantly better survival (p < 0.001), consistent with previous research demonstrating its utility in risk stratification. ^22^

This study has several limitations. First, disease staging was conducted using computed tomography (CT), which may have underestimated the true disease burden compared to positron emission tomography (PET), potentially impacting both staging accuracy and IPI scoring. Second, serum lactate dehydrogenase (LDH) data were unavailable in nine patients, which could reduce the reliability of the calculated IPI scores. Third, this study did not include the assessment of additional immunological markers such as TAM M1 (CD68) and TIL CD4, both of which have also been reported to influence survival outcomes in DLBCL. These limitations were primarily due to constraints in time and funding. Nonetheless, although data collection concluded in early 2022, the clinical relevance of tumor microenvironment markers, such as AMC, CD163, and CD8 has remained prominent in the literature, underscoring the continued applicability of our findings in current oncologic practice.

## Conclusions

Higher expression of tumor-associated macrophages (CD163) and elevated absolute monocyte count (AMC) were significantly associated with poorer two-year event-free survival (EFS) in patients with diffuse large B-cell lymphoma (DLBCL) undergoing R-CHOP therapy. In contrast, increased infiltration of CD8+ tumor-infiltrating lymphocytes (TILs) predicted improved survival outcomes. The optimal prognostic cutoff values identified were 631/mm ^3^ for AMC, 23/HPF for CD163, and 27.5%/HPF for CD8. Moderate correlations were observed between AMC and both tissue-based immune markers, with a positive association with CD163 and a negative association with CD8 expression. These findings support the integration of peripheral and tissue-based immune parameters as complementary tools for risk stratification in DLBCL.

### Authors’ declaration

- We hereby confirm that all the Figures and Tables in the manuscript are ours. Furthermore, any Figures and images, that are not ours, have been included with the necessary permission for re-publication, which is attached to the manuscript.
- Ethical Clearance: The project was approved by the local ethical committee at University of Indonesia.

## Data Availability

Figshare: Prognostic Role of Monocytes, Macrophages, and Lymphocytes in Diffuse Large B-Cell Lymphoma Patients Receiving R-CHOP: A Two-Year Survival Analysis. figshare. Dataset. https://doi.org/10.6084/m9.figshare.29818244.v1 ^29^ The project contains the following underlying data:
• Data.xlsx. (Anonymised data). Data are available under the terms of the Creative Commons Attribution 4.0 International license (CC-BY 4.0).
