# Prognostic Role of Absolute Monocyte Count, CD163⁺ Macrophages, and CD8⁺ Lymphocytes in Diffuse Large B-Cell Lymphoma Treated with R-CHOP

**DOI:** 10.12688/f1000research.168760.2

**Published:** 2026-03-12

**Authors:** Faisal Syarifuddin, Anna Mira Lubis, Agnes Stephanie Harahap, Hamzah Shatri, Wulyo Rajabto, Imam Subekti, Rudy Hidayat, Sukamto Koesnoe

**Affiliations:** 1 Division of Hematology and Medical Oncology, Department of Internal Medicine, Faculty of Medicine, Universitas Indonesia, Rumah Sakit Dr Cipto Mangunkusumo, Central Jakarta, Jakarta, 10430, Indonesia; 2 Department of Pathological Anatomy, Faculty of Medicine, Universitas Indonesia, Rumah Sakit Dr Cipto Mangunkusumo, Central Jakarta, Jakarta, 10430, Indonesia; 3 Division of Psychosomatic and Palliative Medicine, Department of Internal Medicine, Faculty of Medicine, Universitas Indonesia, Rumah Sakit Dr Cipto Mangunkusumo, Central Jakarta, Jakarta, 10430, Indonesia; 4 Division of Endocrinology, Metabolism, and Diabetes, Department of Internal Medicine, Faculty of Medicine, Universitas Indonesia, Rumah Sakit Dr Cipto Mangunkusumo, Central Jakarta, Jakarta, 10430, Indonesia; 5 Division of Rheumatology, Department of Internal Medicine, Faculty of Medicine, Universitas Indonesia, Rumah Sakit Dr Cipto Mangunkusumo, Central Jakarta, Jakarta, 10430, Indonesia; 6 Division of Allergy Immunology, Department of Internal Medicine, Faculty of Medicine, Universitas Indonesia, Rumah Sakit Dr Cipto Mangunkusumo, Central Jakarta, Jakarta, 10430, Indonesia

**Keywords:** DLBCL, monocyte count, CD163, CD8, tumor microenvironment, prognosis

## Abstract

**Background:**

Diffuse large B-cell lymphoma (DLBCL) exhibits heterogeneous clinical outcomes, including variations in event-free survival (EFS). Tumor microenvironment (TME) components, particularly absolute monocyte count (AMC), tumor-associated macrophages (TAMs), and tumor-infiltrating lymphocytes (TILs) have been implicated in prognosis, although findings remain inconsistent. This study evaluates the prognostic value of AMC, TAMs (CD163), and TILs (CD8) on two-year EFS in DLBCL patients treated with R-CHOP.

**Methods:**

A retrospective cohort study of 108 DLBCL patients treated from January 2014 to March 2021 was conducted. AMC was obtained from peripheral blood, while CD163 and CD8 expressions were analyzed via immunohistochemistry. Associations with two-year EFS were assessed using hazard ratios (HR), and correlations between AMC and tissue immune markers were evaluated.

**Results:**

High AMC and CD163 expression were significantly associated with poorer two-year EFS (HR = 9.82 and 8.57; both p < 0.001), whereas elevated CD8 expression predicted better outcomes (HR = 0.13; p < 0.001). AMC positively correlated with CD163 (r = 0.577; p < 0.001) and negatively with CD8 (r = –0.599; p < 0.001).

**Conclusion:**

AMC and CD163 are negative prognostic markers, while CD8 is protective. AMC may reflect the immune profile of the TME and serve as a practical prognostic biomarker in DLBCL.

## Background

Diffuse large B-cell lymphoma (DLBCL) is the most common subtype of non-Hodgkin’s lymphoma, accounting for approximately 30–40% of adult cases. Although the introduction of rituximab-containing immunochemotherapy (R-CHOP) has improved outcomes, clinical prognosis remains heterogeneous, with reported two-year event-free survival (EFS) rates ranging from 39% to 44%. ^1– 3^

Prognostic models such as the International Prognostic Index (IPI)3 and cell-of-origin (COO) classification ^4^ have improved risk stratification but do not fully reflect the biological complexity of DLBCL, particularly the role of host immune responses. ^4, 5^

Increasing evidence highlights the importance of the tumor microenvironment (TME), where immune cells may exert either tumor-promoting or tumor-suppressive effects. ^5, 6^ Among immune components of the TME, tumor-associated macrophages (TAMs), especially CD163-positive macrophages, have been associated with immune evasion and adverse outcomes, whereas CD8-positive tumor-infiltrating lymphocytes (TILs) are generally linked to effective anti-tumor immunity and favorable prognosis. ^5, 7^

However, studies evaluating these markers have reported inconsistent results, likely due to heterogeneity in immunohistochemical methods, cutoff definitions, and survival endpoints. ^5– 9^

Peripheral absolute monocyte count (AMC) has also been proposed as a prognostic biomarker in DLBCL. Elevated AMC may reflect systemic immune dysregulation and contribute to an immunosuppressive tumor microenvironment, although its prognostic value remains debated. ^10, 11^ Integrating peripheral immune parameters with tissue-based immune markers may therefore provide a more comprehensive assessment of host–tumor immune interactions. In addition, event-free survival at 24 months has been proposed as a clinically meaningful surrogate endpoint in DLBCL, as early events strongly correlate with long-term disease-related outcomes. ^12– 14^ Accordingly, this study aimed to evaluate the prognostic impact of absolute monocyte count, CD163-positive tumor-associated macrophages, and CD8-positive tumor-infiltrating lymphocytes on two-year event-free survival in patients with diffuse large B-cell lymphoma treated with R-CHOP, and to explore the relationship between peripheral and tissue-based immune markers.

## Methods

### Study design and setting

This retrospective cohort study analyzed medical record data from patients diagnosed with diffuse large B-cell lymphoma (DLBCL) at Dr. Cipto Mangunkusumo Hospital (RSCM) and the affiliated anatomical pathology laboratory between January 2014 and March 2021. The starting point (time zero) for survival analysis was defined as the date of initiation of first-cycle R-CHOP chemotherapy.

### Ethics statement

This retrospective study was approved by the Health Research Ethics Committee of the Faculty of Medicine, Universitas Indonesia and Cipto Mangunkusumo National Central General Hospital (reference number KET-133/UN2.F1/ETIK/PPM.00.02/2023). Given the retrospective nature of the study, written informed consent was obtained from surviving patients whenever feasible. For patients who had died prior to study initiation, the requirement for informed consent was waived by the ethics committee. Patient confidentiality was strictly maintained, and all data were anonymized prior to analysis.

### Patient selection

**Figure 1.**
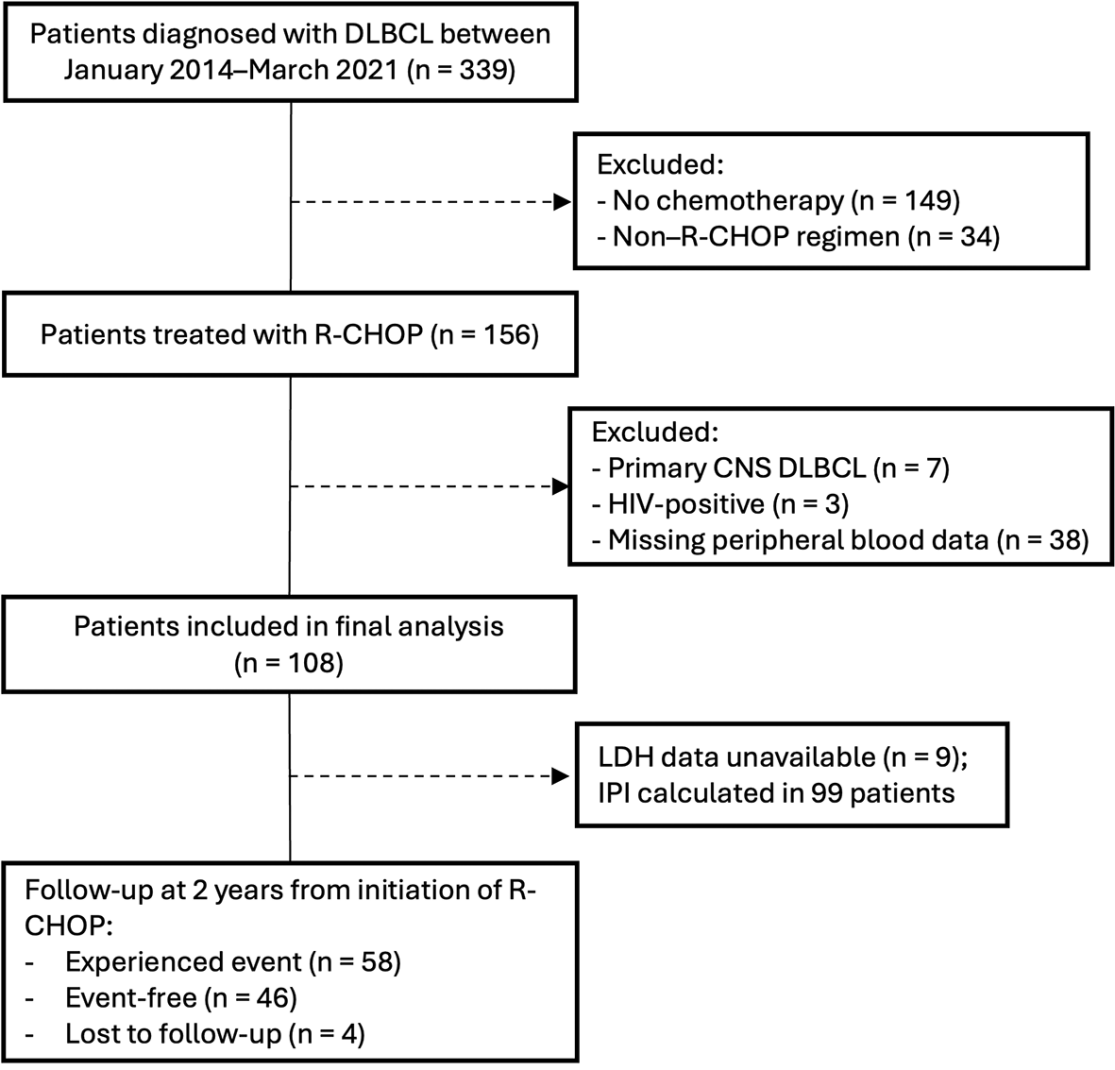
Study flow diagram of patient selection and follow-up.

Inclusion criteria were as follows: (a) age ≥18 years; (b) diagnosis of DLBCL according to the World Health Organization (WHO) classification; (c) receipt of an initial R-CHOP chemotherapy regimen consisting of 6–8 cycles, with or without radiotherapy; and (d) completion of chemotherapy with an average delay of no more than one week per cycle. Exclusion criteria included: (a) a history of indolent lymphoma or evidence of transformed DLBCL (e.g., transformation from follicular lymphoma or chronic lymphocytic leukemia), as these represent biologically distinct entities; (b) discordant or multiple histopathological diagnoses; (c) HIV infection; (d) primary central nervous system (CNS) DLBCL; (e) terminal comorbidities (e.g., end-stage renal failure, decompensated liver cirrhosis, or advanced heart failure); and (f) incomplete clinical or pathological data. Patient status was confirmed via telephone contact using contact information recorded in the medical charts. Baseline clinical characteristics collected included demographic data, number of extranodal sites, Ann Arbor stage, serum lactate dehydrogenase (LDH) levels, International Prognostic Index (IPI) score, and cell-of-origin classification based on the Hans algorithm. A flow diagram illustrating patient identification, exclusion criteria, and final cohort assembly is presented in Figure 1.

### Peripheral blood analysis

Absolute monocyte count (AMC) was obtained from peripheral blood samples analyzed using a Sysmex XN-1000 or XN-3000 hematology analyzer (Sysmex Corporation, Kobe, Japan).

### Immunohistochemical staining

Formalin-fixed, paraffin-embedded (FFPE) tissue blocks were sectioned at 3–4 μm and mounted on poly-L-lysine–coated slides. Sections were deparaffinized in xylene and rehydrated through a graded alcohol series. Antigen retrieval was performed using Tris–EDTA buffer (pH 9.0) in a decloaking chamber at 98°C for 10 minutes, followed by cooling and washing with phosphate-buffered saline (PBS). Endogenous peroxidase activity was blocked using SeyTek Super Block. Slides were incubated for 30 minutes with Bond™ ready-to-use primary antibodies against CD163 and CD8 (Biocare Medical, Netherlands). After washing, sections were treated with a Val Universal Linker secondary antibody and visualized using a high-contrast diaminobenzidine (DAB) substrate. Counterstaining was performed with Mayer’s hematoxylin, followed by bluing in lithium carbonate and dehydration through graded alcohol and xylene. Slides were coverslipped using entellan. Negative controls were included in each staining batch, while tonsil tissue served as a positive control. For quantitative analysis, five representative high-power fields (HPFs; 400× magnification, area 0.24 mm ^2^) were photographed using a Leica ICC50HD microscope with a camera attachment. Quantification of CD163-positive macrophages and CD8-positive lymphocytes was performed using ImageJ ^®^ software. CD163 expression was quantified as the number of positively stained macrophages per high-power field (cells/HPF). CD8 expression was quantified as the percentage of CD8-positive lymphocytes among total nucleated cells within each high-power field (%). Staining intensity was not incorporated into the analysis to reduce variability related to tissue fixation and processing. All evaluations were independently performed by two blinded investigators to ensure inter-observer reliability.

### Event-free survival (EFS)

Event-free survival (EFS) was defined as the time from initiation of R-CHOP chemotherapy to the first occurrence of disease progression, relapse after initial response, initiation of second-line therapy, or death from any cause. Patients without an event were censored at the date of last follow-up.

### Statistical analysis

Optimal cutoff values for AMC, CD163, and CD8 were determined using receiver operating characteristic (ROC) curve analysis based on two-year event-free survival. This approach was selected to provide clinically interpretable thresholds while minimizing overfitting in a retrospective cohort. Correlations between AMC, CD163, and CD8 were assessed using Spearman’s correlation test. The median follow-up duration, estimated using a reverse Kaplan–Meier approach, was 20 months. Event-free survival was analyzed using Kaplan–Meier curves and compared using the log-rank test. Univariate and multivariate Cox proportional hazards regression models were employed to estimate hazard ratios (HRs) and 95% confidence intervals (CIs). Variables with a p-value <0.05 in univariate analysis were included in the multivariate model. A two-sided p-value <0.05 was considered statistically significant. Although maximally selected rank statistics have been proposed for determining optimal cutoffs in time-to-event analyses, ROC-based thresholds were selected in this study to provide clinically interpretable values while minimizing model instability in a retrospective cohort with a limited sample size. Future prospective validation using alternative statistical approaches, including maximally selected rank statistics or time-dependent ROC methods, would be valuable. All statistical analyses were performed using SPSS version 20.0 (IBM Corp., Armonk, NY, USA). Missing data were handled using a complete-case analysis approach; variables with missing values were excluded from analyses requiring those parameters, and no data imputation was performed. All data were independently verified by two reviewers to ensure accuracy and completeness.

## Results

### Patient characteristics

Between January 2014 and March 2021, a total of 108 patients diagnosed with diffuse large B-cell lymphoma (DLBCL) who met the inclusion and exclusion criteria were included in the study. Serum lactate dehydrogenase (LDH) data were missing in nine patients. Among all subjects, 56 (52%) were male, and 72 (66.7%) were younger than 60 years (median age: 53 years; range: 18–88 years). Early-stage disease (Ann Arbor stage I–II) was observed in 60 patients (55.6%), and 89 patients (82%) had 0–1 extranodal sites. Elevated LDH levels (>1× upper limit of normal) were present in 81 patients (81.8%), and 75 patients (75.8%) had a low-risk International Prognostic Index (IPI) score (0–2). Based on the Hans classification, the germinal center B-cell (GCB) subtype was identified in 33 patients (30.6%). During the two-year follow-up period, 58 patients (53.7%) experienced an event, while 46 patients (42.6%) remained event-free. Follow-up information was unavailable for four patients (3.7%). The median values of absolute monocyte count (AMC), CD163 expression, and CD8 expression were 619.2 cells/μL (interquartile range [IQR]: 455.0–759.45), 21.5 cells/HPF (IQR: 14.25–27.0 cells/HPF), and 23% (IQR: 14–39%), respectively. Detailed demographic and clinical characteristics are summarized in Table 1.

**Table 1.** Demographic and clinical characteristics of study subjects.

Variable	Total (%)
**Gender** (n = 108)	
Male	56 (52)
Female	52 (48)
**Age** (Median 53 years, range 18-88 years)	
≤60 years	72 (66,7)
>60 years	36 (33,3)
**Ann Arbor Staging** (n = 108)	
I or II	60 (55,6)
III or IV	48 (44,4)
**Extranodal lesion** (n = 108)	
0-1	89 (82)
>1	19 (18)
**Serum Lactate Dehydrogenase (LDH)** (n = 99)	
≤1 x normal	18 (18,2)
>1 x normal	81 (81,8)
**IPI Score** (n = 99)	
Low risk (0-2)	75 (75,8)
High risk (3-5)	24 (24,2)
**Protein expression** (n = 108)	
CD8, Median (IQR)	0,23 (0,14-0,39)
CD163, Median (IQR)	21,5 (14,25-27,0)
AMC (n = 108), Median (IQR)	619,2 (455,0-759,45)
**Subtypes based on Hans algorithm** (n = 108)	
GCB	33 (30.6)
Non-GCB	75 (69,4)
**Events in 2 years** (n = 108)	
Yes	58 (53,7)
No	46 (42,6)
No Information	4 (3,7)

### Immunohistological characteristics and optimal cutoff values for AMC, CD163, and CD8

Receiver operating characteristic (ROC) curve analysis was performed to determine optimal cutoff values for AMC, CD163, and CD8 in predicting two-year event-free survival (EFS). The optimal cutoff for AMC was 631 cells/μL, yielding an area under the curve (AUC) of 0.923 (95% CI: 0.866–0.981), with a sensitivity of 82.8% and specificity of 90.0%. For CD163 expression, the optimal cutoff was 23 cells/HPF, with an AUC of 0.931 (95% CI: 0.880–0.983), sensitivity of 75.9%, and specificity of 96.0%. CD8 expression demonstrated an optimal cutoff value of 27.5%, with an AUC of 0.852 (95% CI: 0.773–0.930), sensitivity of 84.5%, and specificity of 84.0%.

**Figure 2.**
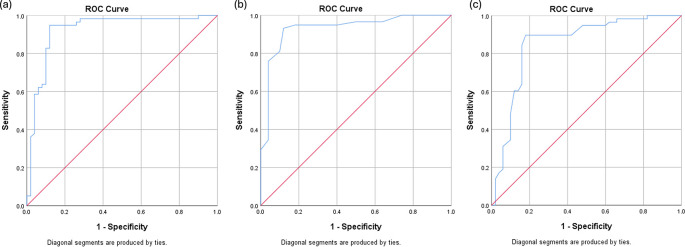
Quantitative ROC curves for 2-year Event-Free Survival: (a) AMC, (b) TAM CD163, and (c) TIL CD8.

**Figure 3.**
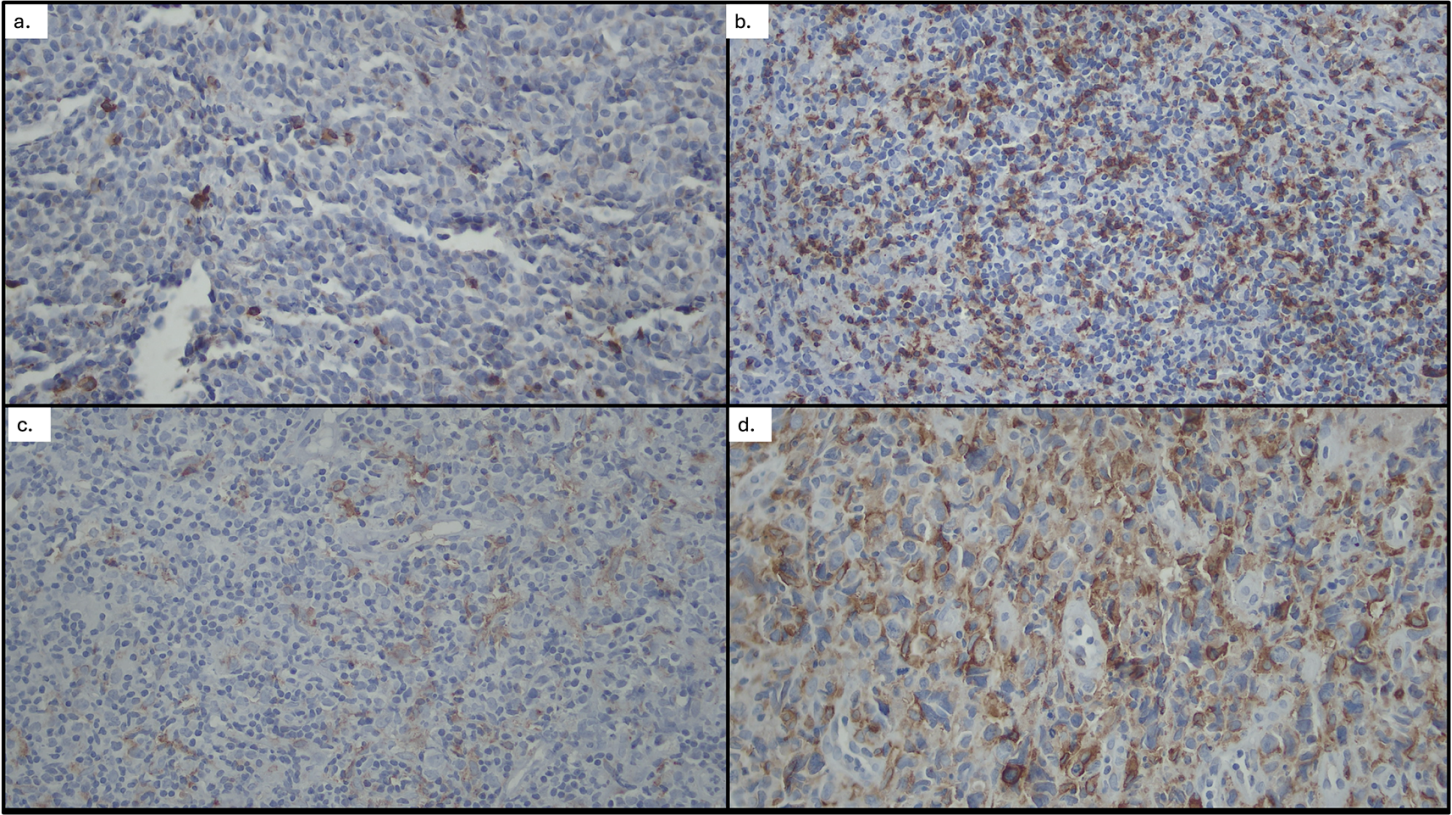
CD163 and CD8 expression in DLBCL tissues (100×): (a) Low CD163 expression, (b) High CD163 expression, (c) High CD8 expression, (d) Low CD8 expression.

ROC curves are presented in Figure 2. Patients were subsequently categorized into high- and low-expression groups according to these cutoff values ( Figure 3).

### Correlation between AMC and CD163 or CD8 expression

**Figure 4.**
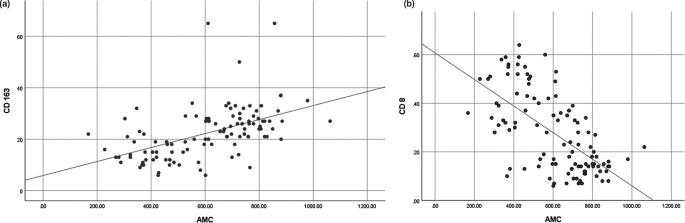
Correlation between (a) AMC and TAM CD163, and (b) AMC and TIL CD8.

Spearman’s correlation analysis demonstrated a significant positive correlation between AMC and CD163 expression (r = 0.577, p < 0.001) and a significant negative correlation between AMC and CD8 expression (r = −0.599, p < 0.001). These findings suggest a potential association between peripheral monocyte levels and the immune composition of the tumor microenvironment. Correlation analyses are illustrated in Figure 4.

### Impact of AMC, CD163, and CD8 on 2-Year EFS

**Figure 5.**
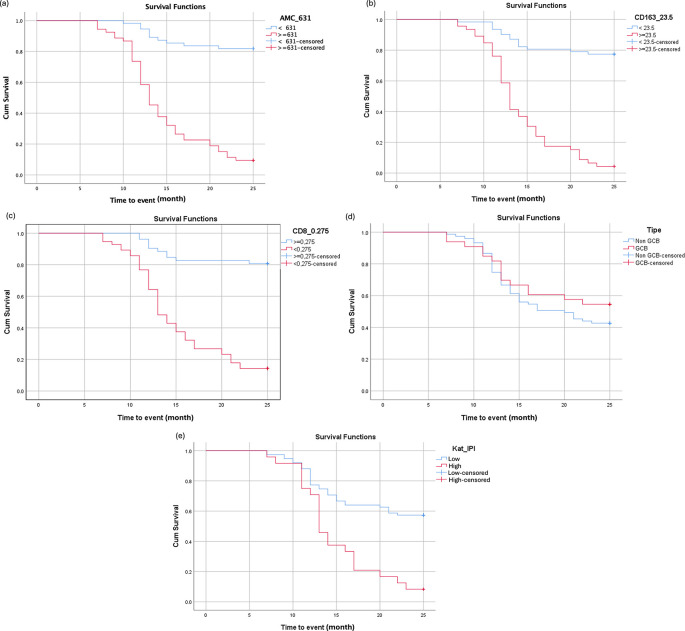
Kaplan-Meier survival curves for DLBCL patients based on (a) AMC, (b) TAM CD163, (c) TIL CD8, (d) COO, and (e) IPI score.

Patients with high AMC levels (≥631 cells/μL) had significantly poorer two-year EFS compared with those with lower AMC levels (HR = 9.82; 95% CI: 4.89–19.70; p < 0.001). Similarly, elevated CD163 expression (≥23 cells/HPF) was associated with inferior EFS outcomes (HR = 8.57; 95% CI: 4.58–16.05; p < 0.001). In contrast, patients with higher CD8 expression (≥27.5%) demonstrated significantly improved EFS (HR = 0.13; 95% CI: 0.06–0.26; p < 0.001). Subgroup analysis based on the Hans cell-of-origin classification showed no significant difference in EFS between the GCB and non-GCB subtypes (HR = 0.75; 95% CI: 0.42–1.35; p = 0.336). In contrast, a high IPI score was significantly associated with reduced EFS (HR = 3.06; 95% CI: 1.77–5.35; p < 0.001). Detailed survival analyses are presented in Table 2 and Figure 5. The median event-free survival time for the overall cohort was 13 months.

**Table 2.** The relationship of different variables on patient survival.

Variable	Event		HR (CI 95%)	p
	Yes (%)	No (%)		
**AMC**				
≥631	48 (90,6)	5 (9,4)	9,817 (4,891-19,703)	<0,001
<631	10 (18,2)	45 (81,8)		
**CD163**				
>23	44 (95,7)	2 (4,3)	8,571 (4,576-16,053)	<0,0001
<23	14 (22,6)	48 (77,4)		
**CD8**				
≥0,275	9 (19,2)	48 (80,8)	0,128 (0.064-0.256)	<0,001
<0,275	48 (85,7)	8 (14,3)		
**GCB/nonGCB**				
GCB	15 (45,5)	18 (54,5)	0,749 (0,416-1,349)	0,336
nonGCB	43 (57,3)	32 (42,7)		
**IPI**				
High	22 (91,7)	2 (8,3)	3,075 (1,768-5,349)	<0,001
Low	32 (42,7)	43 (57,3)		

## Discussion

This study evaluated the prognostic significance of peripheral absolute monocyte count (AMC), CD163-positive tumor-associated macrophages, and CD8-positive tumor-infiltrating lymphocytes in patients with diffuse large B-cell lymphoma treated with R-CHOP. We demonstrated that elevated AMC and increased CD163 expression were significantly associated with inferior two-year event-free survival, whereas higher CD8 infiltration predicted improved outcomes. These findings support the concept that both systemic immune parameters and local tumor microenvironment composition contribute to disease progression in DLBCL. The median age of 53 years in our cohort is consistent with prior Indonesian studies but lower than that reported in Western and East Asian populations, where median ages typically range from the early to mid-60s. ^2, 9, 14^ This difference may reflect variations in genetic background, environmental factors, and infectious cofactors such as Epstein–Barr virus. Additionally, the predominance of early-stage disease in our cohort may partly relate to staging practices and limited access to positron emission tomography in resource-constrained settings. ^15, 16^

More than half of the patients experienced an event within two years of treatment initiation, consistent with previous reports indicating that approximately 40–50% of patients with DLBCL experience relapse or treatment failure despite standard immunochemotherapy. ^17, 18^

In this context, early event-free survival has been recognized as a clinically meaningful surrogate endpoint, as events occurring within 24 months strongly correlate with long-term disease-related mortality. ^12^ ^–^ ^14^ Because event-free survival in this study incorporated death from any cause as an event, a competing-risk framework was not applied. However, future analyses differentiating disease-specific progression from non–disease-related mortality using competing-risk methods may provide additional granularity in prognostic modeling.

Therefore, identifying accessible biomarkers that predict early events remains highly relevant in clinical practice. Elevated peripheral monocyte count has previously been associated with inferior survival outcomes in DLBCL, potentially reflecting systemic inflammation and monocyte recruitment to the tumor microenvironment. ^11, 19^ Our findings are consistent with this hypothesis, as AMC demonstrated both a strong adverse prognostic impact and a moderate positive correlation with CD163 expression. CD163-positive macrophages represent an immunosuppressive M2-polarized phenotype, which has been implicated in tumor immune evasion, angiogenesis, and resistance to therapy. ^19– 21^

Although cutoff values for CD163 vary across studies, methodological differences in staining techniques, quantification methods, and tissue sampling likely account for this variability.

Conversely, higher CD8-positive T-cell infiltration was associated with improved event-free survival, reinforcing the protective role of cytotoxic lymphocytes in mediating anti-tumor immunity. ^7^ The higher CD8 threshold observed in our study compared with some prior reports may reflect the use of whole-section analysis rather than tissue microarrays, which can influence quantification of tumor-infiltrating lymphocytes. ^22– 24^

Notably, the inverse correlation between AMC and CD8 expression suggests a potential imbalance between immunosuppressive macrophage activity and cytotoxic immune responses, underscoring the dynamic and context-dependent interplay within the DLBCL tumor microenvironment. ^25, 26^ The cell-of-origin classification based on the Hans algorithm did not significantly influence two-year event-free survival in our cohort, consistent with several studies questioning its prognostic reproducibility when assessed by immunohistochemistry alone. ^20, 27, 28^

In contrast, the International Prognostic Index remained a robust predictor of outcome, reaffirming its clinical utility in risk stratification. These findings suggest that immune-based biomarkers may provide complementary biological insight beyond conventional clinical scoring systems.

Several limitations should be acknowledged. First, disease staging was based on computed tomography rather than positron emission tomography, which may underestimate disease burden and influence risk stratification. Second, serum lactate dehydrogenase data were unavailable in a subset of patients, potentially affecting IPI calculation. Third, additional immune markers such as CD68-positive macrophages or CD4-positive lymphocytes were not assessed due to resource constraints. Finally, the retrospective design introduces the possibility of selection bias and residual confounding. Nevertheless, the consistency of our findings with prior literature supports the relevance of integrated peripheral and tissue-based immune profiling in DLBCL. In addition, the optimal cutoff values were derived from a single-center cohort using ROC-based thresholds, which may be susceptible to optimism bias. Therefore, external validation in independent cohorts is necessary to confirm the robustness and generalizability of these prognostic thresholds.

## Conclusions

Higher expression of tumor-associated macrophages (CD163) and elevated absolute monocyte count (AMC) were significantly associated with poorer two-year event-free survival (EFS) in patients with diffuse large B-cell lymphoma (DLBCL) undergoing R-CHOP therapy. In contrast, increased infiltration of CD8+ tumor-infiltrating lymphocytes (TILs) predicted improved survival outcomes. The optimal prognostic cutoff values identified were 631/mm ^3^ for AMC, 23/HPF for CD163, and 27.5%/HPF for CD8. Moderate correlations were observed between AMC and both tissue-based immune markers, with a positive association with CD163 and a negative association with CD8 expression. These findings support the integration of peripheral and tissue-based immune parameters as complementary tools for risk stratification in DLBCL.

### Authors’ declaration

- We hereby confirm that all the Figures and Tables in the manuscript are ours. Furthermore, any Figures and images, that are not ours, have been included with the necessary permission for re-publication, which is attached to the manuscript.
- Ethical Clearance: The project was approved by the local ethical committee at University of Indonesia.

## Data Availability

Figshare: Prognostic Role of Monocytes, Macrophages, and Lymphocytes in Diffuse Large B-Cell Lymphoma Patients Receiving R-CHOP: A Two-Year Survival Analysis. figshare. Dataset. https://doi.org/10.6084/m9.figshare.29818244.v1 ^29^ The project contains the following underlying data:
• Data.xlsx. (Anonymised data). Data are available under the terms of the Creative Commons Attribution 4.0 International license (CC-BY 4.0).
