# Supplementary material for: Prognostic Role of Absolute Monocyte Count, CD163⁺ Macrophages, and CD8⁺ Lymphocytes in Diffuse Large B-Cell Lymphoma Treated with R-CHOP
Source: F1000Res. 2026 Mar 12;14:814. Originally published 2025 Aug 22. [Version 2] doi: 10.12688/f1000research.168760.2 (PMC13036440; doi:10.12688/f1000research.168760.2)
Supplement: Peer review history

# Reviewer report: Reviewer response for version 2

**DOI:** 10.5256/f1000research.196782.r467762

**Published:** 2026-04-06

**Authors:** Teng Fei

**Affiliations:** 1 Memorial Sloan Kettering Cancer Center, New York, USA

The authors applied statistical methods to assess the prognostic values of AMC, CD163 and CD8 markers for the event-free survival of DLBCL patients treated with R-CHOP. As a statistical reviewer, I would like to point out the following comments and suggestions. To summarize, flaws are observed in the statistical analysis involved in this manuscript, which should be fully addressed to be considered for indexing.

1. Please justify why it was 2-year EFS that was used to determine the thresholds for the markers of interest.
2. Please use the wording of “univariable” and “multivariable” regression models, rather than “univariate” and “multivariate”. This would comply with the reporting standard.
3. Some approaches such as maxstat and time-dependent ROC were mentioned in the method section but not applied for the analyses. Please move the corresponding description to the discussion section as future directions.
4. Table 1: the title for the second column (Total (%)) is not applicable for the continuous variables.
5. Figure 4: please make the two panels the same size with the same scale for the x-axis.
6. Table 2 is confusing. First, it is unclear whether it reports univariable models or multivariable models. Second, it is untypical to report contingency tables for survival models. After checking the sum of numbers from cells of contingency tables, I suppose the results are univariable (108 patients with AMC, but only 99 with ICI). It is then questionable why univariable results, rather than the multivariable ones, are reported to support the main conclusions of this manuscript.
7. It is stated in the method section that the median follow-up was 20 months. However, no censored observations were reflected in Kaplan-Meier curves from Figure 5, which is contradictory. In addition, there appears to be a strong immortal bias in the population, where all patients survived beyond 6 months, which might be the case but is generally a red flag in statistical review.
8. Event times are undefined for censored patients in the shared data. I assume the authors used administrative censoring at the end of 2 years, but that would contradict the median follow-up of 20 months.

Is the work clearly and accurately presented and does it cite the current literature?

Partly

If applicable, is the statistical analysis and its interpretation appropriate?

No

Are all the source data underlying the results available to ensure full reproducibility?

No

Is the study design appropriate and is the work technically sound?

No

Are the conclusions drawn adequately supported by the results?

No

Are sufficient details of methods and analysis provided to allow replication by others?

Yes

Reviewer Expertise:

Biostatistics

I confirm that I have read this submission and believe that I have an appropriate level of expertise to state that I do not consider it to be of an acceptable scientific standard, for reasons outlined above.

# Reviewer report: Reviewer response for version 2

**DOI:** 10.5256/f1000research.196782.r467144

**Published:** 2026-03-30

**Authors:** Rafael Pichardo-Rodriguez

**Affiliations:** 1 Instituto de Investigaciones en Ciencias Biomédicas (INICIB), Universidad Ricardo Palma, Lima, Peru

I have no further comments; I congratulate the authors on their effort.

Is the work clearly and accurately presented and does it cite the current literature?

Yes

If applicable, is the statistical analysis and its interpretation appropriate?

Partly

Are all the source data underlying the results available to ensure full reproducibility?

Yes

Is the study design appropriate and is the work technically sound?

No

Are the conclusions drawn adequately supported by the results?

No

Are sufficient details of methods and analysis provided to allow replication by others?

Yes

Reviewer Expertise:

Hemato-Oncology, Lymphoma.

I confirm that I have read this submission and believe that I have an appropriate level of expertise to confirm that it is of an acceptable scientific standard.

# Reviewer report: Reviewer response for version 1

**DOI:** 10.5256/f1000research.185964.r430460

**Published:** 2025-11-20

**Authors:** Rafael Pichardo-Rodriguez

**Affiliations:** 1 Instituto de Investigaciones en Ciencias Biomédicas (INICIB), Universidad Ricardo Palma, Lima, Peru

Thank you for the opportunity to review this manuscript, which evaluates prognostic biomarkers in diffuse large B-cell lymphoma. The study addresses a clinically relevant and timely topic; however, there are several methodological and reporting issues that should be addressed before the manuscript can be considered for indexing. My specific comments are as follows:

1. **Operational definition of EFS**
  The Methods section does not specify the operational definition of *event-free survival (EFS)*. While EFS is an increasingly used endpoint in lymphoma, its interpretation requires caution since it is a heterogeneous surrogate outcome. I recommend that the authors clearly define EFS and consider, in subsequent analyses, a **competing-risk approach** to differentiate disease progression/recurrence from non–disease-related mortality.
2. **Estimation and reporting of median follow-up**
  The manuscript does not describe how the median follow-up time was estimated, nor does it report the actual value. This parameter is essential to determine whether the observation period was long enough to capture meaningful clinical events. The authors should specify the method used (e.g., *reverse Kaplan–Meier*) and include the corresponding median follow-up.
3. **Flow diagram (CONSORT/REMARK)**
  A patient flow diagram is not provided. Such a diagram is crucial to visualize the selection process, exclusions, and losses to follow-up. The authors should include a flowchart following the **CONSORT** or **STROBE **guidelines as appropriate.
4. **Definition of follow-up period and time zero**
  The starting point ( *time zero*) for follow-up is not defined. Clarifying whether this corresponds to the date of diagnosis, initiation of treatment, or completion of therapy is critical for reproducibility and for appropriate interpretation of time-to-event analyses.
5. **Handling of missing data**
  The manuscript does not mention whether missing data were present or how they were handled. The authors should state whether they performed data imputation, complete-case analysis, or any other strategy, as this can influence the validity of their findings.
6. **Exclusion criteria**
  The exclusion of “indolent lymphomas” is mentioned, yet this group would not typically be included in a cohort restricted to DLBCL. Instead, it would be more relevant to specify whether **transformed DLBCL cases (e.g., from CLL or follicular lymphoma)** were excluded, since these represent a distinct biological entity.
7. **Determination of cut-off values for continuous biomarkers**
  There is no standardized methodology for defining cut-off points of continuous biomarkers in DLBCL. For time-dependent outcomes, I recommend using **Maximally Selected Rank Statistics** with the **maxstat** package in R to determine optimal thresholds, although this is left to the authors’ discretion.

In summary, this manuscript investigates clinically meaningful and novel biomarkers in Lymphoma. With the suggested methodological clarifications and improvements, the study could be considerably strengthened and would merit reconsideration in a second review round.

 Thank you again for the opportunity to evaluate this valuable work, and I commend the authors for their contribution to the field of lymphoma research

Is the work clearly and accurately presented and does it cite the current literature?

Yes

If applicable, is the statistical analysis and its interpretation appropriate?

Partly

Are all the source data underlying the results available to ensure full reproducibility?

Yes

Is the study design appropriate and is the work technically sound?

No

Are the conclusions drawn adequately supported by the results?

No

Are sufficient details of methods and analysis provided to allow replication by others?

Yes

Reviewer Expertise:

Hemato-Oncology, Lymphoma.

I confirm that I have read this submission and believe that I have an appropriate level of expertise to confirm that it is of an acceptable scientific standard, however I have significant reservations, as outlined above.

# Response

**Published:** 2026-02-21

**Authors:** Faisal Syarifuddin

**Affiliations:** Internal medicine, Faculty of Medicine, University of Indonesia, Jakarta, DKI Jakarta, Indonesia

We sincerely thank the reviewer for the thorough and insightful methodological evaluation of our manuscript. We greatly appreciate the constructive recommendations, which have substantially improved the clarity, transparency, and rigor of our study. The revisions are summarized below:

- The operational definition of event-free survival (EFS) has now been explicitly defined in the Methods section as the time from initiation of first-cycle R-CHOP chemotherapy to the first occurrence of disease progression, relapse after response, initiation of second-line therapy, or death from any cause. We also clarified the rationale for selecting two-year EFS as a clinically meaningful endpoint based on established literature. Additionally, we have acknowledged in the Discussion that future studies may benefit from competing-risk modeling to distinguish disease-specific progression from non–disease-related mortality.
- The median follow-up duration is now reported (20 months), estimated using the reverse Kaplan–Meier method. The method used for estimation has been explicitly stated in the Statistical Analysis section.
- A patient flow diagram has been added (Figure 1) to transparently illustrate patient identification, exclusions, and final cohort assembly, in alignment with STROBE/REMARK recommendations.
- The starting point (time zero) for follow-up has been clearly defined as the initiation date of first-cycle R-CHOP chemotherapy to ensure reproducibility and appropriate interpretation of time-to-event analyses.
- Handling of missing data has now been explicitly described. A complete-case analysis approach was used, and no data imputation was performed. The extent of missing LDH data is now reported in the Results section.
- The exclusion criteria have been clarified to explicitly state that transformed DLBCL cases (e.g., transformation from follicular lymphoma or chronic lymphocytic leukemia) were excluded, as these represent biologically distinct entities.
- Regarding the determination of cut-off values for continuous biomarkers, we have clarified that receiver operating characteristic (ROC) analysis based on two-year EFS was selected to provide clinically interpretable thresholds while minimizing model instability in a retrospective cohort of limited sample size. We have acknowledged in the Statistical Analysis and Discussion sections that alternative approaches, such as maximally selected rank statistics, may be considered in future prospective validation studies.
- The limitations section has been expanded to acknowledge the single-center design, potential optimism bias associated with ROC-derived thresholds, and the need for external validation in independent cohorts.

We are grateful for the reviewer’s careful assessment and believe that these revisions have substantially strengthened the methodological transparency and overall quality of the manuscript.

# Reviewer report: Reviewer response for version 1

**DOI:** 10.5256/f1000research.185964.r427565

**Published:** 2025-11-12

**Authors:** Jenifer Vaughan

**Affiliations:** 1 University of the Witwatersrand J, Johannesburg, Gauteng, South Africa

This is a well written article which has assessed the prognostic influence of elevated peripheral blood monocyte counts along with  CD163 expressing macrophages and CD8+ lymphocytes within the tumour microenvironment of DLBCL. The findings recapitulate those of previous studies, and while they are not novel, they add to the body of literature in this regard. There are however, a few areas which could be improved upon:

  

1. The title is somewhat misleading, as the study focused on specific subsets of macrophages and lymphocytes, and not on all macrophages and lymphocytes. It would be better to specify that CD163+ macrophages and CD8+ lymphocytes were evaluated This should be applied in the abstract as well.
2. It is stated that all of the participants gave written informed consent, but this was a retrospective study, and some of the participants had demised before the study commenced. Please clarify how this was possible.
3. Under the immunohistochemical staining methods, it is stated that CD8 was reported as a percentage of stained T-cells. How were all the T-cells identified if only a CD8 stain was used?
4. There is an error in the 1 ^st^ paragraph of the Results, where it is stated that four patients remained event free (it should be 46 patients according to Table 1).
5. In the following line of the same paragraph, there is an error in the number of numbers of CD163 positive cells (reported as a percentage and not cells/HPF).
6. In Table 1, units should be included for the numbers provided for the AMC, CD8 and CD163.
7. There is an error in the legend for Figure 2; it is stated that (d) shows low CD8 expression, but the image appears to show high CD8 expression?
8. Why was 2 year EFS looked at and not the more overall meaningful survival statistic of overall survival?
9. In the 2 ^nd^ paragraph of the Discussion, it is stated that Extranodal involvement was seen in 27% of patients. This information is not included in the Results section?
10. Similarly, in the 4 ^th^ paragraph of the Discussion, it is stated that the median survival time was 13 months, but this too was not included in the Results section. It is not a good practice to introduce new results in the Discussion.

Is the work clearly and accurately presented and does it cite the current literature?

Yes

If applicable, is the statistical analysis and its interpretation appropriate?

Yes

Are all the source data underlying the results available to ensure full reproducibility?

Yes

Is the study design appropriate and is the work technically sound?

Yes

Are the conclusions drawn adequately supported by the results?

Yes

Are sufficient details of methods and analysis provided to allow replication by others?

Partly

Reviewer Expertise:

Tumour microenvironement of DLBCL, HIV-associated lymphoma

I confirm that I have read this submission and believe that I have an appropriate level of expertise to confirm that it is of an acceptable scientific standard.

# Response

**Published:** 2026-02-21

**Authors:** Faisal Syarifuddin

**Affiliations:** Internal medicine, Faculty of Medicine, University of Indonesia, Jakarta, DKI Jakarta, Indonesia

We sincerely thank the reviewer for the constructive feedback, which has significantly improved the clarity and methodological transparency of our manuscript. The revisions are summarized below:

- The title has been revised to explicitly specify CD163⁺ macrophages and CD8⁺ lymphocytes to accurately reflect the immune subsets evaluated. Corresponding changes have been made in the Abstract for consistency.
- The Ethics section has been clarified to state that written informed consent was obtained from surviving patients whenever feasible, while consent for deceased patients was waived in accordance with ethics committee approval.
- The immunohistochemical quantification method has been revised to clarify that CD8 expression was measured as the percentage of CD8-positive lymphocytes among total nucleated cells within each high-power field using ImageJ® software, correcting the previous ambiguity.
- The typographical error regarding the number of event-free patients has been corrected (46 patients). The unit for CD163 expression has been standardized as cells/HPF throughout the manuscript.
- Units for AMC (cells/µL), CD163 (cells/HPF), and CD8 (%) have been added in Table 1 to improve clarity.
- The figure legend has been corrected to accurately reflect CD8 expression levels.
- Extranodal involvement and the median event-free survival time (13 months) are now explicitly reported in the Results section to avoid introducing new findings in the Discussion.
- The rationale for selecting two-year event-free survival (EFS24) as the primary endpoint has been clarified, emphasizing its established relevance as a clinically meaningful surrogate marker in DLBCL.

We greatly appreciate the reviewer’s thoughtful comments, which have strengthened the overall quality and transparency of the manuscript.
